# Knowledge, Attitudes, and Practices About Zoonotic Diseases in Livestock Producers From Three Municipalities of Magdalena Medio, Antioquia

**DOI:** 10.1155/vmi/3399047

**Published:** 2025-11-06

**Authors:** Licet Paola Molina-Guzmán, Leonardo Alberto Ríos-Osorio, Lina Andrea Gutiérrez-Builes, Jaiberth Antonio Cardona-Arias

**Affiliations:** ^1^Escuela de Ciencias de la Salud, Facultad de Medicina, Grupo Biología de Sistemas, Universidad Pontificia Bolivariana, Medellín, Colombia; ^2^Escuela de Microbiología, Grupo de Investigación Salud y Sostenibilidad, Universidad de Antioquia, Medellín, Colombia

**Keywords:** attitudes, knowledge, livestock producers, practices, zoonoses

## Abstract

**Background:**

Zoonotic diseases related to cattle farming cause a significant sanitary and economic impact in Colombia. Poor knowledge, negative attitudes, and few practices related to their transmission, prevention, and control aggravate the epidemiological profile of these diseases.

**Objective:**

To analyze the profile of knowledge, attitudes, and practices (KAP) related to zoonotic diseases in livestock producers from three municipalities of Magdalena Medio de Antioquia.

**Methods:**

A cross-sectional study of 143 randomly selected livestock producers who participated voluntarily. A reproducible and valid survey was applied to estimate KAP scores; the description was made with frequencies and summary measures. The factors associated with KAP were determined with nonparametric tests. Potential explanatory factors were identified with multivariate linear regressions.

**Results:**

Most of the subjects were young men from rural areas with middle socioeconomic status, married, employed in general farm maintenance, and with low formal education. The main factors associated with the KAP profile were area of residence, gender, marital status, age, and time working in the activity. Knowledge about vectors of infectious agents was deficient in relation to the attitudes domain. It was found that they receive little information, and in the evaluation of practices, they are at high risk concerning the lack of protective equipment and consumption of untreated water and raw food.

**Conclusions:**

Education in the promotion and maintenance of health, as well as veterinary supervision in the livestock production setting, are central factors for preventing zoonotic diseases. This study generates a valid scale for monitoring and research associated with cattle farming.

## 1. Introduction

Zoonoses are infectious diseases transmissible from vertebrates to humans, with a wide diversity of causal agents including viruses, bacteria, parasites, fungi, and prions [1]. They comprise approximately 60% of all known infectious diseases; it is estimated that 75% of emerging infectious agents are zoonotic [2].

The incidence of emerging zoonotic diseases has increased since the 1940s, despite efforts to control them, due to demographic changes, alterations in land use, urbanization, increased travel and trade, agricultural practices, and encroachment on natural habitats [3].

Zoonotic pathogens substantially affect the economy and public health in terms of morbidity and decreased livestock productivity, which is more severe in low-income countries [4]. This is worsened by the fact that domestic livestock play an important role as a potential source of new zoonotic pathogens. In this regard, Mesquita [5] found that ungulates are the most important nonhuman hosts in terms of the number of emerging and re-emerging zoonotic pathogen species that can infect different host classes, which facilitates transmission, colonization, and infection of different host species [5].

In this context, it should be kept in mind that transmission between livestock and humans occurs predominantly in agricultural workers (among whom livestock producers stand out); research and educational actions in this group represent a unique opportunity to address several components of the transmission of this type of disease. In this line of thought, previous studies carried out in Magdalena Medio and Norte de Antioquia have been mainly based on the description of risk factors [6], prevalence of infection [7], and vector distribution [8], with little research investigating knowledge, attitudes, and practices (KAP) about zoonotic diseases.

KAP studies are based on several premises, among which stands out the fact that behaviors regarding a given topic are largely determined by the level of knowledge (information or cognitive structures) on that topic, as well as the attitudes (favorable or unfavorable feelings or emotions) of people toward it. Therefore, in cases where it is desired to enhance protective practices or mitigate risky behaviors, it is necessary to know KAP, since knowledge and attitudes are predictors of effective practices [9].

In that sense, several KAP studies on zoonotic diseases are available in the world literature and have highlighted that these surveys provide crucial information to explore risk factors and possible intervention strategies for disease management [10]. Livestock farmers' behavior is strongly affected by their knowledge and attitudes [11], and poor knowledge of a disease correlates with its high prevalence and can trigger a vicious cycle between underdiagnosis/underreporting and awareness deficit [12]. Likewise, some studies have mentioned that the evaluation and description of KAPs among livestock producers about the risk of zoonotic diseases can help develop and implement disease prevention and control strategies in the livestock population, as well as the development and implementation of more efficient community-based health education. Therefore, this study aimed to analyze the KAP on zoonotic diseases in livestock producers in three municipalities of Magdalena Medio de Antioquia.

## 2. Materials and Methods

### 2.1. Study Area

**Figure 1:**
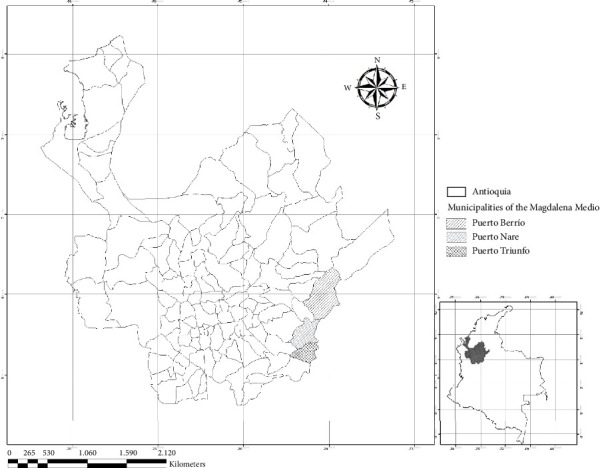
Geographical location of the three municipalities of Magdalena Medio of Antioquia included in the study.

Cross-sectional and psychometric evaluation of a scale applied to workers from 48 cattle farms in Magdalena Medio, a subregion of Antioquia, specifically in the municipalities of Puerto Berrío, Puerto Nare, and Puerto Triunfo (Figure 1).

### 2.2. Study Subjects

To calculate the sample size for the research work, a universe of 11,202 people dedicated to livestock farming was taken into account, and a random selection of 143 subjects was made who met the following inclusion criteria: personnel of any gender, of legal age, and who were in direct contact with the reference animal population (technical and veterinary personnel, zootechnicians, agronomists and agricultural technicians, livestock farmers, and workers). The exclusion criteria were people with mental disorders that could generate a loss of information due to memory bias and those who refused to participate in the study during the collection of information.

### 2.3. KAP Scale

To select the items of the three dimensions (knowledge, attitudes, and practices) of the KAP scale, a literature review and consultation with experts were carried out. The experts were a bacteriologist with a PhD in Basic Biomedical Sciences, and 6 years of experience in the study of some zoonotic diseases associated with livestock production; a specialist in Basic Biomedical Sciences, and PhD in Sustainability, Technology, and Humanism, with experience in the diagnosis and study of the resilience of diseases associated with different production systems; a veterinarian with experience in the management and technical assistance in livestock production systems; a biologist with experience in the diagnosis of some diseases associated with cattle production; a member of the most representative associations of livestock production systems in Antioquia, with more than 10 years of experience in cattle production, management of diseases associated with the system and notification reporting to animal health systems; and an epidemiologist with experience in scale design and psychometric studies. This was initially evaluated under the criterion of logical or appearance validity, carried out by the experts consulted for its construction to determine its applicability, and with 10 people from the target population to determine its acceptability.

In total, 25 items were included to evaluate knowledge, 13 for attitudes and 9 for practices, these were measured in three levels (1 being the lowest and 3 the highest), at the end a sum was made, so that the higher scores showed better KAP against zoonotic diseases. The scores of the three dimensions were unified on a scale from 0 (*worst*) to 100 (*best*) with the following formula [(score obtained in the initial sum − minimum possible score)/Range of the initial score] ∗ 100.

### 2.4. Collection of Information

A primary source of information was used, which included a survey completed by interviewers trained in the contents of the instrument, techniques for approaching the respondent, ethical aspects, privacy conditions that the place where the surveys were answered must meet, and the review of the survey before it was handed over for filing. The survey included sociodemographic variables, family and personal history, and a survey to evaluate KAPs concerning zoonotic diseases.

### 2.5. Bias Control

The interviewers were trained. The participation of the study subjects was motivated by the presentation of the objectives, ethical aspects, and other components of the project. A pilot test was carried out, and a measurement scale was applied with appearance, content, predictive, and reproducible validity in the criteria of reliability, internal consistency, and discriminant power.

### 2.6. Statistical Analysis

The description of the study group was made with frequencies and summary measures. The content validity of the three dimensions of the KAP scale was done by means of exploratory factor analysis, taking as satisfactory those items with *λ* coefficients (factor loadings) greater than 0.30. For predictive validity, the percentage of variance explained by the items of each dimension was estimated.

Three properties were evaluated for reproducibility: (i) reliability with Cronbach's alpha (satisfactory for values ≥ 0.7); (ii) internal consistency using Spearman's rank correlation coefficients item—dimension to which it belongs (satisfactory ≥ 0.40); and (iii) discriminant power using Spearman's rank correlation coefficients item—dimension to which it does not belong (satisfactory if it was lower than the correlation found in the internal consistency). Construct validity was evaluated using exploratory factor analysis, estimating factor loadings and goodness of fit with the Kaiser–Meyer–Olkin test and Bartlett's sphericity.

The KAP scores were described with summary measures, their association with baseline characteristics of the study group was explored using nonparametric tests given the noncompliance with the bivariate normality assumption (assessed with Shapiro–Wilk), and potential explanatory factors were identified with multivariate linear regressions for each KAP score. Analyses were performed in SPSS 29.0®, with a significance of 0.05.

### 2.7. Ethical Aspects

The guidelines of the Declaration of Helsinki and Resolution 8430 of 1993 from the Colombian Ministry of Health were followed. The project was approved by the Research Ethics Committee of Universidad Pontificia Bolivariana (Minute No. 7).

## 3. Results

### 3.1. Description of the Study Population

Most of the study subjects were male, from rural areas, of middle socioeconomic status, married or in a common-law marriage, and with a job in general maintenance. 50% were between 20 and 42 years old, and 50% had passed only 5 or fewer years of schooling. The median time working with cattle was 20 years, and they reported at least 19 days per month spent on this work (Table 1).

### 3.2. Description of KAP Scale Items

Table 2 presents the factor loadings of each item of the three KAP dimensions. Favorable results for content validity are observed, insofar as all the items presented *λ* coefficients greater than 0.30.

In the psychometric evaluation, excellent properties of reproducibility with reliability were found. The percentage of success was greater than 85% in internal consistency and greater than 90% in discriminant power. In predictive validity, explained variance percentages between 30% and 52% were found, while in content validity the success rate was greater than 85%, with favorable results in goodness of fit. Overall, the psychometric properties presented above demonstrate the relevance of the KAP construct (Table 3).

It is worth clarifying that the psychometric performance would be 100% successful with the exclusion of the item “Some ticks, flies or fleas transmit zoonotic diseases” in the Knowledge dimension, the items “If you know a person has a zoonosis, would you stay away from them?” and “It is normal for people to have a zoonosis” in attitudes and “Wash hands after daily work or contact with animals” in practices.

KAP scores did not show statistical differences among the municipalities evaluated (Kruskal–Wallis p H > 0.05), socioeconomic status (Kruskal–Wallis p H > 0.05), years of study (Spearman's rho *p* > 0.05), or time worked on the farm (Spearman's rho *p* > 0.05).

Table 4 shows some factors associated with KAP scores; the knowledge presented statistically significant differences with the area of residence (lower in rural territory), profession (lower in people in charge of general herd maintenance), and years in the activity (lower in those with fewer years of experience). The practices showed statistical differences related to gender, marital status, age, years of experience, and time working in livestock activities.

### 3.3. Potential Explanatory Factors for KAP Scores

The linear regression models identified the potential explanatory factors of the KAP scores; for the knowledge score, they were the location of residence, being 11 points higher in people living in urban areas, and the time working in this type of activity, with an increase of 0.7 points in knowledge for each increase of one year of work. Attitudes were based on socioeconomic status (better in the lower status), years of study (better in people with more schooling), and time working on this activity (better in those who have been working in this activity for fewer years). Practice dimension was better in older people. In addition, the regression model demonstrated convergence of KAP scores, particularly corroborating that practices in this population are related to the levels of knowledge and attitudes (Table 5).

## 4. Discussion

In this study, a KAP scale was designed and validated, which showed excellent reproducibility and validity. In the reproducibility breakdown, satisfactory results were found in reliability, internal consistency, and discriminant power, demonstrating that the scale items present a good degree of uniformity for each domain. Each dimension includes questions that are coherent among themselves and different from the two additional dimensions. Likewise, validity evidenced that the construct and factorial structure of the KAP scale was adequate for the selected items.

Findings on knowledge were associated with area of residence and years of experience, being lower in rural and novice producers, similar to findings from a recent study from Iran that assessed the KAP of small ruminant farmers and found an association between experience and understanding with respect to several infectious diseases [13]. Similarly, in a recent study assessing zoonotic disease risks among livestock farmers in smallholder communities in Ethiopia, those interviewed who never attended school were three times more likely to answer zoonosis-related questions correctly than those who did [11]. Conversely, a recent study assessing the knowledge and opinion of dairy farmers in Malaysia indicated that those with higher education had a better understanding of zoonoses [14].

Moutos et al. [15] indicated that the level of disease awareness among North American producers is associated with disease prevalence, as well as high risk due to a lack of knowledge of ways of transmission [15]. In this study, cattle producers did not recognize ticks, flies, or fleas as possible vectors transmitting infectious agents of zoonotic diseases. They also presented a lack of knowledge of the zoonotic nature of some agents, despite a significant prevalence documented in farms [10, 16–19].

Studies in South Africa [10, 11] and India [12] have shown that schooling plays a crucial role in producers' knowledge of zoonoses. In addition, the absence of collaboration with a veterinarian has also been linked to low knowledge and risky practices for zoonosis transmission [20]. Our findings indicate that, in relation to attitudes, years of study (better in people with more schooling), time working in livestock activities, and people with more age and more time working in activities related to cattle were associated with better practices and knowledge for the prevention of zoonotic diseases.

In the practices, some high-risk practices were identified, such as the lack of use of protective equipment and the consumption of untreated water and raw food, the most relevant. This coincides with previous studies on zoonoses, in which it has been reported that these can be transmitted to humans through contaminated milk, meat, air, feed or contact with infected animals, but this fact is not known by all farmers, who are documented to have a high proportion of subjects with these types of risk practices, such as handling biological material without protective equipment and consuming raw foods such as cheese from unpasteurized milk, raw milk, or food without adequate or sufficient cooking [12, 21, 22]. From the results obtained, it could be suggested that high-risk self-reported practices imply a knowledge gap about zoonotic disease transmission and the prevalence of zoonotic diseases.

The present study has several limitations, both observational and cross-sectional. Given the cross-sectional nature of the present survey, it is not possible to document causal relationships. In addition, considering that the analysis was based on questionnaires, there is a possibility that recall bias may have occurred. Additionally, response bias is unavoidable due to the use of multiple-choice answers, although it could have been mitigated in the construction of the knowledge score due to some strict requirements. Attitudinal responses clearly do not correspond to practices, because many farmers admitted that, despite knowing the appropriate action, they do not implement it either because of the cost or the manual labor required. This is even more understandable considering the high-risk behaviors discovered through self-reported practices. In addition, the findings have weak external validity (generalizability) due to convenience and local sampling. However, Cronbach's alpha results suggest that the completed questionnaire demonstrated good internal consistency.

The study's findings align closely with recent literature from the past 5 years, reinforcing key trends in zoonotic disease awareness and behaviors among livestock producers. Participants demonstrated limited knowledge regarding transmission vectors such as ticks, flies, and fleas, which is consistent with findings from Ngoshe et al. [10] in South Africa and Acharya et al. [17] in South Korea, both of which highlighted occupational exposure combined with poor vector recognition.

Despite reporting high attitude and practice scores, this study revealed persistent high-risk behaviors, such as the consumption of untreated water and raw animal products, as well as the inadequate use of personal protective equipment. This disconnect between awareness and actual practices has been similarly observed in the works of Sadiq et al. [14] and Alemayehu et al. [11], suggesting that positive attitudes do not necessarily translate into safe behaviors without structural or educational reinforcement.

Sociodemographic factors—including urban or rural residence, education level, and years of experience—were statistically associated with variations in KAP dimensions, echoing the findings of Moutos et al. [15] and Kusumaningrum et al. [9], who emphasized the importance of access to veterinary and public health services in shaping zoonotic awareness and prevention.

Finally, the psychometric strength of the validated KAP scale used in this study, with Cronbach's alpha values exceeding 0.86 for both knowledge and practice dimensions, supports its utility for future research and surveillance initiatives. This aligns with recommendations from Tazerji et al. [3], who underscore the need for standardized, context-specific instruments to monitor zoonotic risks and guide One Health interventions.

This study revealed significant gaps in KAP regarding zoonotic diseases among livestock producers in Puerto Berrío, Puerto Nare, and Puerto Triunfo. Although participants scored high on attitudes (average = 73) and especially on practices (average = 100), their knowledge scores were notably lower (average = 64), with many failing to recognize transmission vectors such as ticks, flies, or fleas. Psychometric validation confirmed the KAP scale's reliability (Cronbach's alpha: knowledge = 0.896; practices = 0.872), supporting its use in research and public health efforts. Regression analysis showed that knowledge was significantly higher among urban residents (+11 points) and those with more years in the sector (+0.66 points per year). Attitudes improved with higher education levels and were unexpectedly better among those with fewer years in the field. Practices were strongly associated with older age and were positively linked to higher knowledge and attitude scores, demonstrating convergent validity among the KAP components. Alarmingly, unsafe practices—such as not using personal protective equipment and consuming raw or unboiled products—continued despite positive self-reported attitudes, indicating a gap between knowledge and behavior. For example, although 100% of participants reported using safe practices, many still engaged in risky behaviors when responses were broken down by item. Based on these findings, we recommend:

Implementing targeted educational programs to address critical knowledge gaps, particularly in rural areas where scores were significantly lower (urban mean = 76 vs. rural = 62; *p*=0.002).

Strengthening veterinary extension services and promoting continuous farmer engagement, especially with low-education or maintenance-role workers who had lower knowledge scores (mean = 60).

Adopting One Health-based community education policies, focusing on both individual behavior change and systemic support.

## Figures and Tables

**Table 1:** Description of the study population.

		*n*	%
Municipality	Puerto Berrio	50	35.0
	Puerto Triunfo	37	25.9
	Puerto Nare	56	39.2
Gender	Male	118	82.5
	Female	25	17.5
Residence area	Urban	24	16.8
	Rural	119	83.2
Socioeconomic status	Lower (1–2)	58	40.6
	Middle (3–4)	62	43.4
	Upper (5–6)	23	16.1
Marital status	Single	38	26.6
	Married—common-law marriage	102	71.3
	Divorced	3	2.1
Profession	Technician	12	8.5
	Maintenance	103	73.0
	Administrator	26	18.4

	**Me (IR)**	***X*** ± **SD**	**Min–Max**

Age	42 (32–50)	43 ± 13	20–85
Years of study	5 (3–7)	5 ± 4	0–17
Years working with cattle	20 (10–28)	19.5 ± 11.7	1–50
Years on current farm	1.3 (0–5)	4.2 ± 7	0–45
Years in this activity	0 (0–0)	1.5 ± 7	0–50
Hours per day	8 (1–8)	5 ± 4	0–14
Days per week	6 (0–8)	5 ± 3	0–15
Days per month	24 (4–30)	19 ± 13	0–30

*Note:* Me (IR): median with interquartile range. *X* ± SD: mean with standard deviation. Min–max: range, minimum to maximum.

**Table 2:** Description of KAP scale items.

Knowledge	*λ* coefficients
Symptoms	
Zoonoses can cause skin outbreaks	0.95
Zoonoses can cause headaches	0.92
Zoonoses can cause fatigue	0.92
Zoonoses can cause joint pain	0.91
Zoonoses can cause fever	0.88
Way of transmission	
A person can contract zoonosis through contact with cattle	0.83
A person can contract zoonosis from drinking raw milk	0.83
A person can contract zoonosis through contact with rodents	0.80
A person can contract zoonosis through work practices	0.79
A person can contract zoonosis through contact with wild animals	0.78
Some zoonoses can be contracted through direct contact with cattle body fluids (blood, semen, saliva, urine, and fecal matter)	0.78
Do you know how zoonotic diseases are transmitted?	0.69
Some zoonoses can be contracted from walking barefoot	0.62
Zoonotic diseases can be transmitted from one person to another	0.41
Some ticks, flies, or fleas transmit zoonotic diseases	0.59
Risk groups	
Pregnant women are at high risk of contracting zoonoses	0.85
Elderly people are at high risk of contracting zoonoses	0.83
Children are at high risk of contracting zoonoses	0.76
Workers are at high risk of contracting zoonoses	0.66
People can have a zoonosis in their body and not be sick	0.36
Zoonosis control	
Do you know how to diagnose zoonosis?	0.84
Do you know how to treat zoonosis?	0.75
Do you know what zoonoses are or something about them?	0.64
Zoonoses can be diagnosed in the laboratory	0.51
Some zoonoses can cause death	0.48
Attitudes	
If you were diagnosed with zoonosis, would you have the treatment recommended by your doctor?	0.71
Workers should change their work clothes every time they work before going home	0.68
If you have repeated fevers, would you go to the doctor?	0.67
If you were diagnosed with zoonosis, would you go to the hospital for check-ups?	0.66
If you know a person has a zoonosis, would you stay away from them?	0.66
Once a year workers should receive information about zoonotic diseases	0.61
At least once a year, people should have a laboratory test for exposure to zoonoses	0.59
In all houses, people in contact with animals should check their bodies after work	0.55
If you knew you had a zoonosis, would you stop working?	0.54
Would anything happen to me by being in the same house or space with someone who has a zoonosis?	0.50
In all houses, people should wash their hands before eating	0.50
It is normal for people to have a zoonosis	0.41
If you were diagnosed with zoonosis, would you treat it at home?	0.38
Practices	
Training on zoonosis helps prevent transmission	0.81
Living with different animals allows zoonoses to spread	0.76
Wearing boots in the field protects against animal or insect bites	0.76
Wash hands after daily work or contact with animals	0.73
When an animal dies or breeds, the remains of the body or placenta should be buried	0.68
Pest control at home and in the workplace helps prevent zoonosis	0.62
Eating uncooked food may help disease transmission	0.60
Consumption of boiled or drinking water can help to prevent transmission of zoonoses	0.60
Wearing gloves, boots, and long shirts when working with cattle should be necessary	0.34

**Table 3:** Psychometric evaluation and description of the KAP scale.

	Knowledge	Attitudes	Practices
Reliability			
Cronbach's alpha	0.896	0.680	0.872
Internal consistency			
Spearman's rho rank	0.37–0.71	0.12–0.50	0.37–0.70
Success rate	96 (24/25)	85 (11/13)	89 (8/9)
Discriminating power			
Spearman's rho rank	0.10–0.29	0.00–0.26	0.04–0.28
Success rate	100 (50/50)	100 (26/26)	100 (18/18)
Predictive validity			
Explained variance (%)	35	30	52
Content validity			
*λ* Rank	0.36–0.95	0.38–0.71	0.34–0.81
Success rate	96 (24/25)	92 (12/13)	89 (8/9)
Goodness of fit			
Kaiser–Meyer–Olkin	0.868	0.811	0.811
Bartlett's sphericity (Vp)	0.001	0.001	0.001
Description of scores			
Median (interquartile range)	61 ± 23	71 ± 15	95 ± 14
Mean ± standard deviation	64 (48–76)	73 (69–77)	100 (100–100)
Minimum–Maximum	4–100	8–92	22–100

**Table 4:** Comparison of KAP scores by population characteristics.

	Knowledge	Attitudes	Practices
	Median (interquartile range)		
Gender			
Male	64 (48–76)	73 (69–77)	100 (100–100)
Female	64 (40–80)	77 (65–77)	100 (89–100)
Mann–Whitney Vp U	0.968	0.963	0.001^∗∗^
Area			
Urban	76 (64–85)	73 (69–81)	100 (94–100)
Rural	62 (46–72)	73 (69–77)	100 (100–100)
Mann–Whitney Vp U	0.002^∗∗^	0.300	0.268
Marital status			
Single	62 (48–84)	69 (65–77)	100 (89–100)
Married—common-law marriage	64 (50–76)	73 (69–81)	100 (100–100)
Mann–Whitney Vp U	0.912	0.116	0.008^∗∗^
Profession			
Technician	85 (76–92)	71 (67–81)	100 (89–100)
Maintenance	60 (44–72)	73 (69–77)	100 (100–100)
Administrator	68 (62–78)	71 (69–81)	100 (100–100)
Kruskal–Wallis Vp	0.001^∗∗^	0.966	0.470

	**Spearman's rho correlation coefficient**		

Age	0.063	−0.063	0.190^∗^
Years working with cattle	−0.045	−0.039	0.175^∗^
Years in this activity	0.234^∗∗^	−0.057	−0.077
Hours per day	−0.024	0.049	0.273^∗∗^
Days per week	−0.072	0.101	0.241^∗∗^
Days per month	−0.050	0.090	0.260^∗∗^

^∗∗^ *p* < 0.01.

^∗^ *p* < 0.05.

**Table 5:** Potential explanatory factors for KAP scores.

	Model variables	Regression coefficient
Knowledge	Residence location (urban/rural)	11.0^∗^
	Years in this activity	0.66^∗∗^
	Practice score	0.58^∗∗^

Attitudes	Socioeconomic status	−2.033^∗∗^
	Years of study approved	0.824^∗∗^
	Time in this activity	−0.479^∗∗^
	Practices	0.324^∗∗^

Practices	Age	0.225^∗∗^
	Knowledge	0.217^∗∗^
	Attitudes	0.295^∗∗^

^∗∗^ *p* < 0.01.

^∗^ *p* < 0.05.

## Data Availability

The authors confirm that the data supporting the findings of this study are available on reasonable request from the corresponding author.
